# Transient heat stress protects from severe endothelial damage and dysfunction during prolonged experimental ex-vivo lung perfusion

**DOI:** 10.3389/fimmu.2024.1390026

**Published:** 2024-05-14

**Authors:** Roumen Parapanov, Anne Debonneville, Manon Allouche, Jérôme Lugrin, Helena Rodriguez-Caro, Lucas Liaudet, Thorsten Krueger

**Affiliations:** ^1^Service of Thoracic Surgery, Lausanne University Hospital, Lausanne, Switzerland; ^2^Service of Adult Intensive Care Medicine, Lausanne University Hospital, Lausanne, Switzerland; ^3^Department of Oncology, University of Lausanne and Ludwig Institute for Cancer Research, Lausanne, Switzerland

**Keywords:** ex-vivo lung perfusion, lung ischemia-reperfusion, pulmonary endothelium, lung transplantation, heat shock response, animal model, heat therapy

## Abstract

**Introduction:**

The pulmonary endothelium is the primary target of lung ischemia-reperfusion injury leading to primary graft dysfunction after lung transplantation. We hypothesized that treating damaged rat lungs by a transient heat stress during ex-vivo lung perfusion (EVLP) to elicit a pulmonary heat shock response could protect the endothelium from severe reperfusion injury.

**Methods:**

Rat lungs damaged by 1h warm ischemia were reperfused on an EVLP platform for up to 6h at a constant temperature (T°) of 37°C (EVLP_37°C_ group), or following a transient heat stress (HS) at 41.5°C from 1 to 1.5h of EVLP (EVLP_HS_ group). A group of lungs exposed to 1h EVLP only (pre-heating conditions) was added as control (Baseline group). In a first protocol, we measured lung heat sock protein expression (HSP70, HSP27 and Hsc70) at selected time-points (n=5/group at each time). In a second protocol, we determined (n=5/group) lung weight gain (edema), pulmonary compliance, oxygenation capacity, pulmonary artery pressure (PAP) and vascular resistance (PVR), the expression of PECAM-1 (CD31) and phosphorylation status of Src-kinase and VE-cadherin in lung tissue, as well as the release in perfusate of cytokines (TNFα, IL-1β) and endothelial biomarkers (sPECAM, von Willebrand Factor -vWF-, sE-selectin and sICAM-1). Histological and immunofluorescent studies assessed perivascular edema and formation of 3-nitrotyrosine (a marker of peroxinitrite) in CD31 lung endothelium.

**Results:**

HS induced an early (3h) and persisting expression of HSP70 and HSP27, without influencing Hsc70. Lungs from the EVLP_37°C_ group developed massive edema, low compliance and oxygenation, elevated PAP and PVR, substantial release of TNFα, IL-1β, s-PECAM, vWF, E-selectin and s-ICAM, as well as significant Src-kinase activation, VE-cadherin phosphorylation, endothelial 3-NT formation and reduced CD31 expression. In marked contrast, all these alterations were either abrogated or significantly attenuated by HS treatment.

**Conclusion:**

The therapeutic application of a transient heat stress during EVLP of damaged rat lungs reduces endothelial permeability, attenuates pulmonary vasoconstriction, prevents src-kinase activation and VE-cadherin phosphorylation, while reducing endothelial peroxinitrite generation and the release of cytokines and endothelial biomarkers. Collectively, these data demonstrate that therapeutic heat stress may represent a promising strategy to protect the lung endothelium from severe reperfusion injury.

## 1 Introduction

Lung transplantation (LTx) is the unique treatment for end-stage lung diseases, but it is limited by the shortage of available lung grafts (1). Ex vivo lung perfusion (EVLP) has been developed to increase the pool of transplantable lungs, by allowing prolonged graft physiological evaluation and for the possible therapeutic reconditioning and repair of damaged donor lungs before LTx (2). As such, EVLP serves the main purpose to alleviate ischemia reperfusion (I/R) injury after LTx, hereby reducing the risk of primary graft dysfunction (PGD) (3). The pulmonary endothelium is a major target of I/R injury during LTx (4). At the onset of reperfusion, pulmonary endothelial cells produce highly reactive oxygen (ROS) and nitrogen species (RNS) which may precipitate endothelial damage and dysfunction, resulting in increased vascular permeability, extravasation of immune cells and lung injury (4–6). Different strategies for pulmonary endothelial protection in the context of lung I/R injury have been evaluated, such as the targeted endothelial delivery of antioxidants (7) or the inhibition of adhesion molecules activities (8), to name a few. Although promising in the experimental setting, their translational application for clinical LTx remains to be established (9).

Previous investigators reported that endothelial cells (ECs) from non-pulmonary origin could be protected against I/R injury through the transient exposure to a mild heat stress (HS) and the induction of a heat-shock response (10, 11). The latter is a highly conserved adaptive mechanism conferring tolerance to a diversity of stressful stimuli, through the induced expression of various heat shock proteins (HSPs), acting as molecular chaperones for the maintenance of proteostasis (12). Proposed mechanisms of HS-dependent endothelial protection in I/R conditions comprise the activation of K_ATP_ channel (10) and the stimulation of NO-mediated vasodilation (11). Furthermore, moderate heat exposure may improve endothelial integrity and function through the expression of various protective genes and the reduction of oxidative stress and inflammation (13, 14). These effects on the systemic endothelium support the hypothesis that heat exposure could also benefit the lung endothelium, particularly in the conditions of endothelial damage and dysfunction associated with I/R injury.

The EVLP platform is ideally suited to address this hypothesis, as it permits to selectively expose the lungs to a precisely calibrated temperature with well-defined time limits. We recently provided evidence that transient heat exposure during EVLP conferred protection against I/R injury of damaged lung grafts, by attenuating lung inflammation, oxidative stress, epithelial damage and physiological deterioration after LTx (15). In the present study, we sought to expand further on the effects of ex-vivo therapeutic heat application, by addressing more specifically its effects on the lung endothelium. Furthermore, since prolonged reperfusion can emphasize the damaging effects of I/R injury on the endothelium (16), the effects of heat exposure were evaluated in an EVLP model with an extended reperfusion time of 6 hours.

## 2 Materials and methods

### 2.1 Animals

Sixty male Sprague-Darley rats weighing 350-400g (Charles River Laboratories, Saint-Germain-Nuelles, France) were used in these experiments. All animals were treated in accordance with the “Guidelines for the Care and Use of Laboratory Animals” (NIH Publication no. 96-23), and the experimental protocol was approved by our local animal committee (authorizations 3456a and 3456x1).

### 2.2 Ex vivo lung perfusion of warm ischemic rat lungs and therapeutic heat exposure

We used our well-established protocol of warm ischemic donor lung procurement and EVLP (15, 17–19). Under general anesthesia (5% isoflurane, followed by Ketamine 80 mg/kg and Xylazine 8 mg/kg i.p.), rats were tracheotomized, mechanically ventilated and sacrificed by exsanguination after systemic anticoagulation with intravenous heparin (600 IU). Animals were kept *in situ* for 1h at room temperature (warm ischemic time). Thereafter, following median sternotomy, the pulmonary artery (PA) and left ventricle were canulated and lungs were flushed with 25 ml of 4°C Perfadex^®^, while ventilated at an FiO2 of 0.5, a respiratory rate (RR) of 25/min, a tidal volume (V_t_) of 7ml/kg and a positive end-expiratory pressure (PEEP) of 3 cm H_2_O. Lungs were then inflated (7ml/kg), the heart-lung block was removed, stored at 4°C in Perfadex ^®^ for 1h (cold ischemic time), weighted and mounted on the EVLP system. For EVLP, perfusion was started at a flow of 2% of estimated cardiac output (CO), then perfusate solution was progressively warmed and flow increased to 7.5% of CO. When perfusate temperature reached 35°C, ventilation was started using a Flexivent FX3 ventilator (SCIREQ Inc., Montréal, Canada) at a V_t_ of 3 ml/kg, a FiO_2_ of 0.21 and a RR of 7/min, increased to V_t_ of 6 ml/kg and RR of 30/min at a perfusate temperature of 37°C.

**Figure 1:**
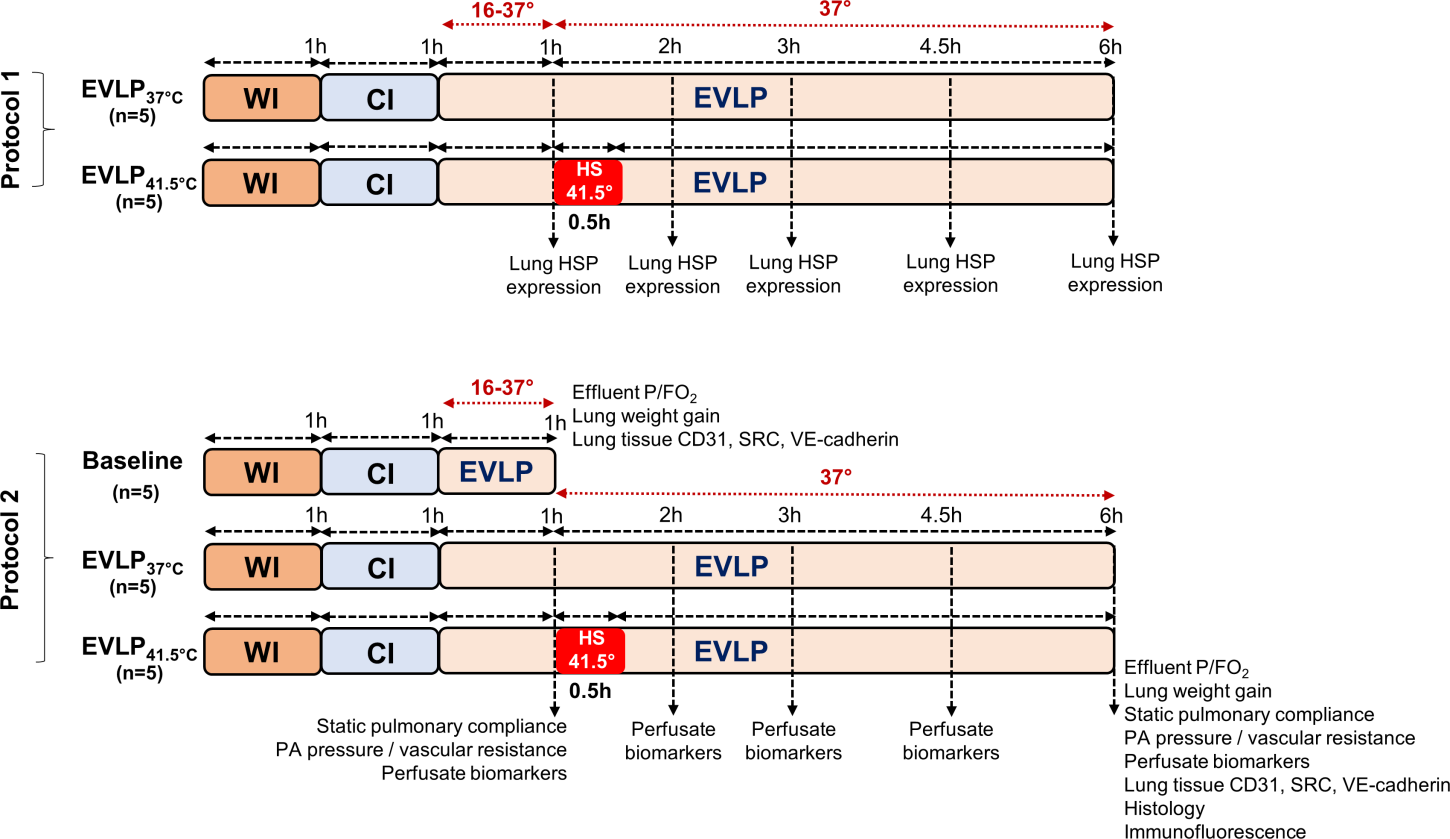
Experimental protocols. In protocol 1, lungs exposed to 1h warm ischemia (WI) and 1h cold ischemia (CI) were maintained on EVLP for 1, 2, 3, 4.5 and 6h, either at constant T° of 37°C (EVLP_37°C_ group) or following transient heat stress at 41.5° for 30 min, starting at 1h EVLP (EVLP_HS_ group). At each time-point, lungs were obtained for HSP expression (n=5/group, except at 1h, n=5 in a single group). In protocol 2, lungs were maintained on EVLP for 1h (Baseline group, pre-heating conditions, n=5) or for 6h, either at constant T° of 37°C (EVLP_37°C_ group, n=5) or following transient heat stress as described above (EVLP_HS_ group, n=5). Measurements performed at the selected time-points are indicated. CI, Cold Ischemia; EVLP, Ex-Vivo Lung Perfusion; HS, Heat Stress; HSPs, Heat Shock Proteins; WI, Warm Ischemia.

The experimental protocols are detailed in **Figure 1**. Lungs were either maintained at a perfusate temperature of 37°C until the end of EVLP (group EVLP_37°C_) or exposed to a transient heat stress (group EVLP_HS_), performed by the rapid warming of perfusate temperature at 41.5°C after 1h EVLP, maintained for 30 minutes and followed by rapid cooling at 37°C until the end of EVLP, as described previously (15). The perfusate temperature was monitored at the entry of the PA with a digital thermometer (TES-1303, Type-K, TES Electrical Electronic Corp.). In a first set of experiments (protocol 1), we evaluated the time-course of heat shock protein induction during EVLP durations of 1, 2, 3, 4.5 and 6h (n=5 animals/time in both groups, except at 1h, where only 1 group of n=5 was evaluated). In a second set of experiments (protocol 2), EVLP was maintained for 6h in n=5 animals from each group (EVLP_37°C_ and EVLP_HS_). In a separate experimental group, EVLP was performed for 1h only (pre-heating, baseline, n=5). At the end of EVLP (1h or 6h), the heart-lung blocks were weighted and the difference between the weight before and after EVLP was computed as an index of lung edema (15, 19, 20). Lungs were then separated, the right lungs were snap-frozen and kept at -80°C for tissue analysis, and the left lungs were fixed in 4% paraformaldehyde and paraffine-embedded for histology and immunofluorescence studies (performed only at the 6h time-point). Samples of perfusates were collected after 1, 2, 3, 4.5 and 6h of EVLP, centrifuged and stored at -80°C, for further biochemical measurements.

### 2.3 Physiological measurements

The static pulmonary compliance (SPC) was determined during EVLP using the Flexivent FX3 Flexiware® software, as previously described (15, 19). The partial pressure of O_2_ was measured at the end of EVLP in the effluent perfusate, using CG4+ cartridge (Abbott i-STAT analyzer, East Windsor, KJ). The PA pressure (PAP) was monitored throughout EVLP and pulmonary vascular resistance (PVR) was calculated as (mean PA pressure-left atrial pressure)/pulmonary flow, expressed in cmH_2_O/ml/min (19). The left arial pressure was maintained at 3 cm H_2_O throughout EVLP.

### 2.4 Biological measurements

#### 2.4.1 Heat shock proteins levels in lung tissue

Lung tissue was powdered in liquid nitrogen, then homogenized in RIPA buffer (0.1% SDS, 1% NP-40, 1% sodium deoxycholate, 150 mM NaCl, 50 mM Tris pH 7.4, 1 mM EDTA), sonicated and incubated for 30 min on ice. The concentrations of the heat shock proteins HSP27, HSP70 (both inducible), and Hsc70 (constitutive) were determined by specific ELISAs (see **Supplementary Table S1**), and expressed in ng/mg tissue protein, measured by the BCA assay (Thermo Scientific Pierce, Rockford, IL, USA).

#### 2.4.2 Western immunoblotting of Src kinase, phospho-Src, VE-cadherin and phospho-VE-cadherin in lung tissue

40 μg of lung proteins were separated on standard SDS-PAGE gels and transferred to nitrocellulose membranes. The blots were then probed with mouse monoclonal anti-Src, rabbit polyclonal anti-phospho-Src (^p^Tyr419 and ^p^Tyr259), rabbit polyclonal anti-VE-cadherin and anti-phospho-VE-cadherin (^p^Tyr685) primary antibodies (**Supplementary Table S1**), and were revealed by chemiluminescence using appropriate secondary antibodies and ECL Western Blotting Reagents (Radiance Plus, Azure biosystems). Beta-actin was used as an internal control. Image acquisition was done with a chemiluminescence camera (Fusion FX®, Vilber, Marne-la-Vallée, France) and the signals were quantified using Image J software.

#### 2.4.3 Inflammatory cytokines and endothelial biomarkers in the EVLP perfusate

Perfusate obtained at selected time-points was assayed for Interleukin-1 beta (IL-1β), tumor necrosis factor alpha (TNF-α), von Willebrand Factor (vWF), soluble E-selectin (sE-selectin), intercellular adhesion molecule-1 (sICAM-1) and platelet endothelial cell adhesion molecule 1 (sPECAM-1), using specific ELISAs (**Supplementary Table S1**). All results are expressed in ng/ml or pg/ml. We also determined the expression of PECAM-1 (CD31) by ELISA in lung tissue homogenates, which was expressed in ng/mg tissue protein.

#### 2.4.4 Histological assessment of perivascular edema

Paraffine-embedded sections (5 µm) were stained with hematoxylin-eosin and digitized using the NDP.view2 image software (Hamatsu Photonics K. K., Shizuoka, Japan). Perivascular edema (arteries and veins) was quantified in 10 fields/section, using a previously published score (21), comprising (a) the number of vessels with perivascular edema (0: absent; 1: mild; 2: moderate; 3: severe), and (b) the thickness of perivascular edema, calculated as a percentage of the inner vessel diameters (0: no edema; 1: < 25%; 2: 25-50%; 3: > 50%). The total score per section was computed as the sum of the (a) and (b) scores in the 10 fields examined. The histological analyses and scoring were performed in a blinded manner by a single investigator (RP).

#### 2.4.5 Immunofluorescence staining of 3-nitrotyrosine and CD31 in lung tissue

Paraffin-embedded tissue sections (5 µm) were dewaxed in xylene and rehydrated. Antigen retrieval was performed at pH 6 before blocking in PBS containing 5% normal donkey serum, 0.1% BSA and 0.3% Triton for 30 minutes. Sections were then incubated simultaneously overnight at 4°C with primary polyclonal goat anti-CD31 antibody (1:100) and primary polyclonal rabbit anti-3-nitrotyrosine (3-NT) antibody (1:1000). After washing in PBS containing 0.3% Triton, sections were incubated 1h at room temperature with secondary antibodies (anti-goat Alexa 555 and anti-rabbit Alexa 647, 1:300) and washed. Coverslips were mounted with Fluoromount-G™ Mounting Medium, with DAPI (ThermoFisher). Negative control sections were incubated only with the secondary antibodies to set the fluorescence threshold. Slides were scanned with NanoZoomer S60 slide-scanner. Fiji (ImageJ, version 1.52h) was used to quantify the percentage of 3-NT area present into CD31 area. The Fiji script used for the analyses is given in the Supplementary information (**Supplementary File S2**). The immunofluorescence analyses and quantification were performed in a blinded manner by a single investigator (AD).

### 2.5 Statistical analysis

All data analyses and statistics were performed with Graphpad prism 10.0.1 (GraphPad Software Inc., La Jolla, CA). Results are presented as means ± SEM unless otherwise specified. Comparisons between groups was done using one-way analysis of variance (ANOVA) followed by Tukey’s test (one single time-point), or two-way ANOVA (multiple time-points), with Tukey *post-hoc* test for the effect of group and Bonferroni adjustment for the effect of time. For comparisons between two groups only (histology), unpaired two-tailed t-test (immunofluorescence studies) or Mann Whitney nonparametric test (histology score) were applied. Statistical significance was assigned to a p value < 0.05.

## 3 Results

### 3.1 Kinetics of protein expression of HSP27, HSP70 and Hsc70 over 6h of EVLP

**Figure 2:**
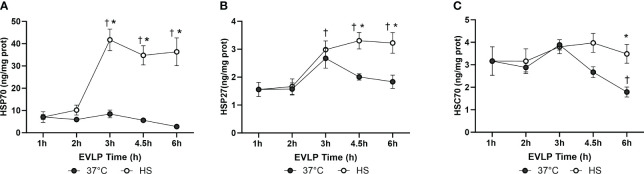
Kinetics of Heat shock protein expression in rat lungs at different time points during EVLP. HSP70 **(A)**, HSP27 **(B)** and HSC70 **(C)** in lung tissue homogenates, expressed in ng/mg lung protein. N=5/group at each time-point, except at 1h (one group only, n=5). * p < 0.05 *vs* EVLP_37°C_; † p < 0.05 *vs* 1h EVLP.

In the EVLP_37°C_ group, the expression levels of inducible HSP70 and HSP27 (**Figures 2A, B**) did not significantly vary over time, whereas the expression of constitutive Hsc70 (**Figure 2C**) significantly dropped after 4.5 and 6h EVLP. In the EVLP_HS_ group, both HSP70 and HSP27 increased significantly after 3h EVLP (that is, 2h after the initiation of heat stress), and then remained at comparable expression values until the end of EVLP, whereas Hsc70 remained unchanged over the 6h EVLP, so that its expression was significantly higher than in EVLP_37°C_ at 4.5 and 6h.

### 3.2 Heat stress reduces lung edema and improves lung physiological function during EVLP

**Figure 3:**
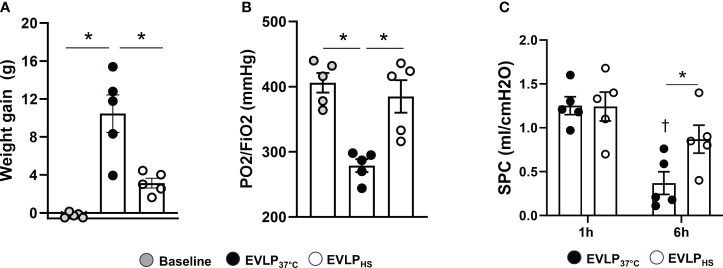
Lung weight gain, oxygenation capacity and static compliance. **(A)** Lung weight gain between the start and the end of EVLP, measured as an index of lung edema, and **(B)** Oxygenation capacity (effluent perfusate P/FO_2_ ratio) were determined after 1h EVLP (Baseline group, n=5) or after 6h EVLP in EVLP_37°C_ and EVLP_HS_ groups (n=5/group). **(C)** Static pulmonary compliance (SPC) was measured after 1 and 6h in EVLP_37°C_ and EVLP_HS_ (n=5/group). * p < 0.05 (inter-group difference); † p < 0.05 (6h *vs* 1h EVLP).

**Figure 4:**
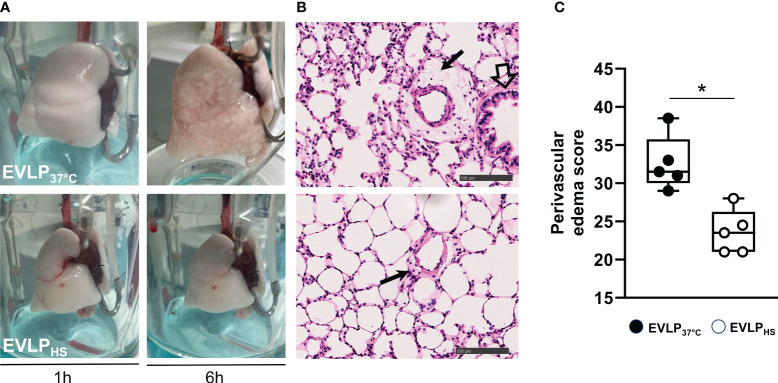
Lung morphology. **(A)** Representative macroscopic pictures of lungs from EVLP_37°C_ (upper images) and EVLP_HS_ (lower images) groups, after 1h and 6h EVLP. **(B)** Representative histological sections of lungs evaluated at 6h of EVLP. The black arrows indicate perivascular edema. The open arrow shows a bronchial structure. **(C)** Score of perivascular lung edema after 6h EVLP. N=5/group. * p < 0.05.

No significant weight gain was noted after 1h EVLP in the baseline group (**Figure 3A**). After 6h EVLP, a major increase in weight was present in the EVLP_37°C_ group, which was considerably less pronounced in the EVLP_HS_ group. Massive lung edema in the EVLP_37°C_ group was associated with a significant reduction of oxygenation capacity (**Figure 3B**) and static pulmonary compliance (**Figure 3C**) after 6h. In contrast, values of both physiological variables remained unaffected in the EVLP_HS_ group. Macroscopic evaluation of the lungs clearly showed the severity of lung edema developing in the EVLP_37°C_ group after 6h and the decrease of such edema in lungs from the EVLP_HS_ group (**Figure 4A**). This was further confirmed by histological analysis of perivascular edema, which was significantly reduced after 6h EVLP in EVLP_HS_ as compared to EVLP_37°C_ (**Figure 4B**, representative sections and 4C, histological score).

### 3.3 Heat stress attenuates the increase of PA pressure and pulmonary vascular resistance during EVLP

**Figure 5:**
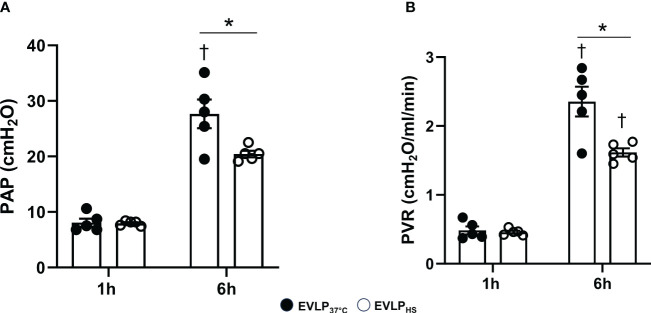
Pulmonary artery pressure and pulmonary vascular resistance during EVLP. **(A)** Pulmonary Artery Pressure and **(B)** Pulmonary Vascular Resistance at 1 and 6h EVLP in EVLP_37°C_ and EVLP_HS_ groups (n=5/group). * p < 0.05 (inter-group difference); † p < 0.05 (6h *vs* 1h EVLP).

PA pressure and PVR after 1h EVLP were comparable between the EVLP_37°C_ and EVLP_HS_ groups (**Figure 5A**). After 6h, a significant increase of PA pressure and PVR was observed in EVLP_37°C_ (**Figure 5B**), and these two changes were significantly attenuated in the EVLP_HS_ group.

### 3.4 Heat stress prevents Src kinase activation and VE-cadherin phosphorylation after 6h EVLP

**Figure 6:**
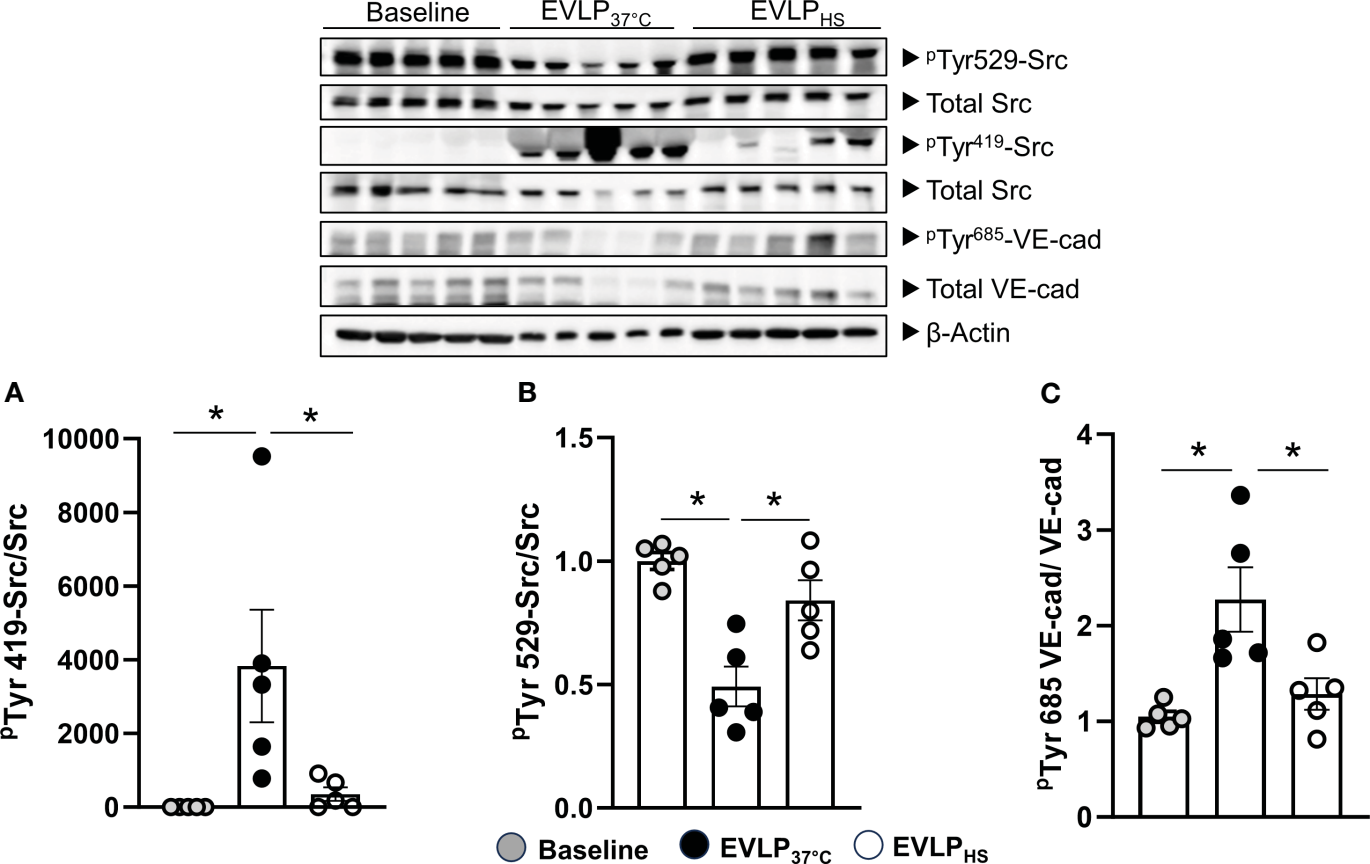
Western immunoblot analysis of Src-kinase and VE-cadherin phosphorylation. Immunoblots (upper panel) and signal quantification **(A–C)** of total Src kinase, ^p^Tyr^419^-Src, ^p^Tyr^529^-Src, total VE-cadherin, ^p^Tyr^685^-VE-cadherin, and β-actin (internal control) determined after 1h EVLP (Baseline group, n=5) or after 6h EVLP in EVLP_37°C_ and EVLP_HS_ groups (n=5/group). ∗p < 0.05.

Owing to the role of Src family kinase in the regulation of endothelial permeability (22), we assessed its activation during EVLP by determining the phosphorylation status of two critical tyrosine residues, namely Tyr^419^ (upregulates Src activity) and Tyr^529^ (inhibits Src activity). In parallel, we determined the phosphorylation status at Tyr^685^ of the cell junction protein VE-cadherin, a key substrate of src kinase. As illustrated in **Figure 6**, Src Tyr^419^ phosphorylation significantly increased, while Src Tyr^529^ phosphorylation (**Figures 6A, B**) significantly decreased in EVLP_37°C_ lungs in comparison to the baseline group (1h EVLP), and these changes were associated with significant Tyr^685^ phosphorylation of VE-cadherin (**Figure 6C**). In marked contrast, the phosphorylation status of Src kinase and VE-cadherin were unchanged in the EVLP_HS_ lungs, as compared to baseline.

### 3.5 Heat stress reduces the formation of 3-nitrotyrosine in endothelial cells after 6h EVLP

**Figure 7:**
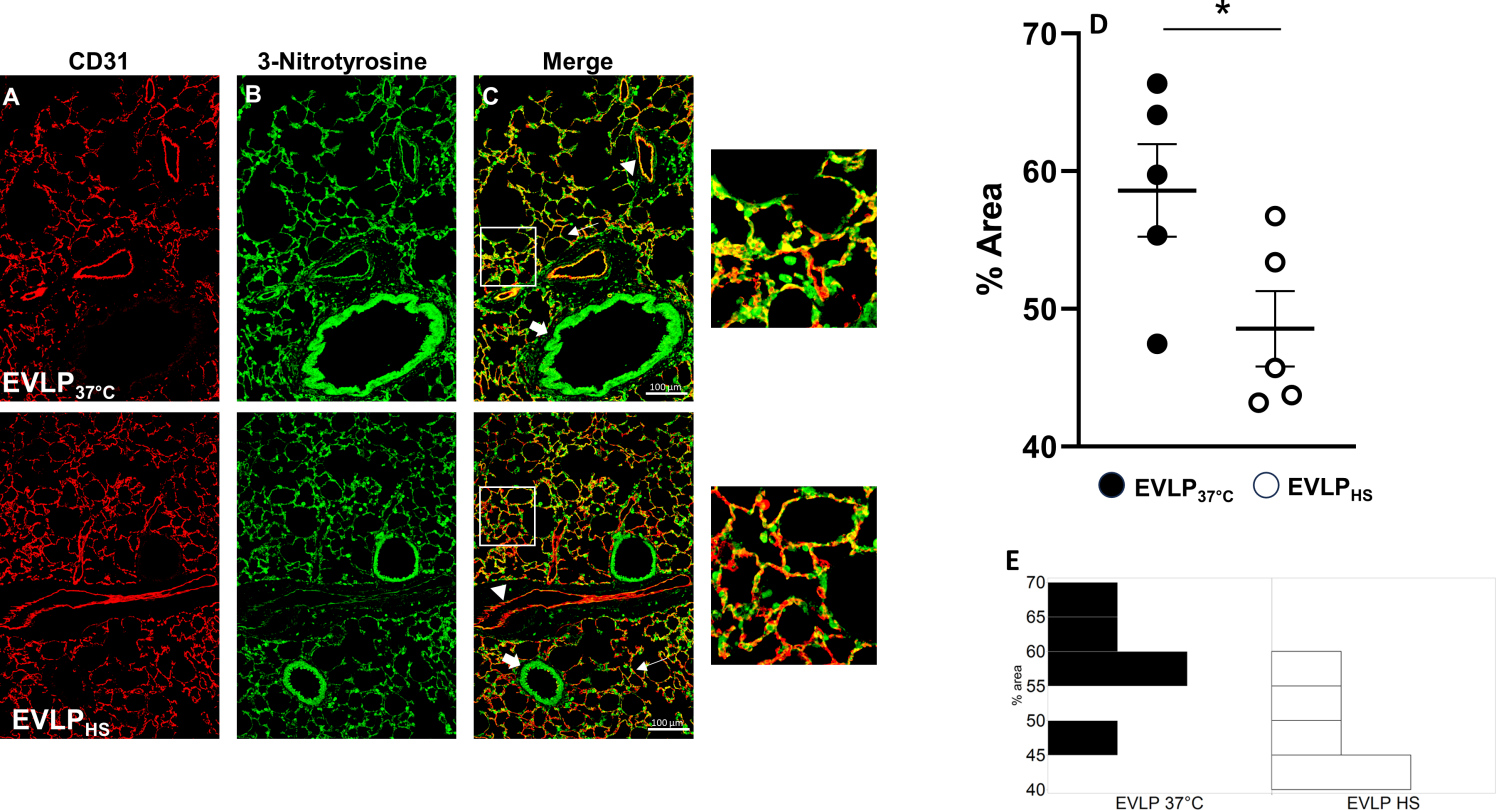
Immunofluorescent detection of 3-nitrotyrosine and CD31. Representative sections of lungs from the EVLP_37°C_ (upper images) and EVLP_HS_ (lower images) groups after 6h EVLP. **(A)** CD31 immunostaining; **(B)** 3-NT immunostaining; **(C)** Merged images from **(A, B)** The regions encircled in the white squares in **(C)** are shown at higher magnification on the right. White arrows: alveolar structures; Arrowheads: extra-alveolar vessels; Large arrows: bronchioles. **(D)** Quantification of immunofluorescence staining of 3-NT formation in CD31 endothelium. **(E)** Histogram of the distribution of the data in each group. N=5/group. *p < 0.05.

As illustrated in **Figures 7A–C**, both EVLP_37°C_ and EVLP_HS_ lungs displayed positive 3-NT immunostaining in the endothelial lining of alveolar and extra-alveolar vessels, as labeled with anti-CD31 antibody, as well as in the wall of small bronchioles. The quantified intensity of endothelial 3-NT staining was significantly greater in lungs from the EVLP_37°C_ group (**Figure 7D**), which further displayed thicker vessel walls than lungs from the EVLP_HS_ group as highlighted in the insert of **Figure 7C**. **Figure 7E** shows an histogram of the distribution of the data in each group.

### 3.6 Heat stress alleviates the release of inflammatory cytokines and endothelial biomarkers in the perfusate during EVLP

**Figure 8:**
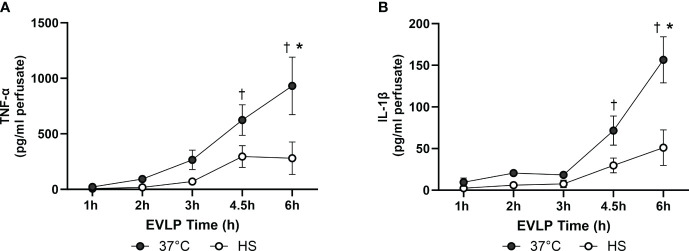
Levels of inflammatory cytokines in the EVLP perfusate. **(A)** TNF-α and **(B)** IL-1β, measured in the EVLP perfusate at different time-points in the EVLP_37°C_ and EVLP_HS_ groups. N =5/group. * p < 0.05 *vs* EVLP_37°C_; † p < 0.05 *vs* 1h EVLP.

**Figure 9:**
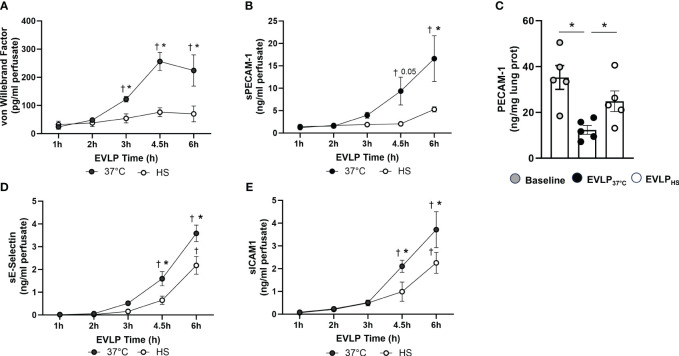
Release of endothelial biomarkers during EVLP. **(A)** Von Willebrand Factor and **(B)** Soluble PECAM-1, measured at selected time-points in the perfusate from EVLP_37°C_ and EVLP_HS_ groups. **(C)** Lung tissue expression of PECAM-1 determined after 1h EVLP (baseline group) and after 6h EVLP in the EVLP_37°C_ and EVLP_HS_ groups. **(D, E)** Perfusate levels of Soluble E-selectin **(D)** and Soluble ICAM-1 **(E)** in EVLP_37°C_ and EVLP_HS_ groups. N=5/group. * p < 0.05 (intergroup differences); † p < 0.05 *vs* 1h EVLP.

As shown in **Figures 8A, B**, a progressive increase in the perfusate content of TNFα and IL-1β occurred in the EVLP_37°C_ group, an effect significantly prevented by heat stress. Furthermore, as illustrated in **Figures 9A, B**, a significant release of vWF and s-PECAM was detected in the EVLP_37°C_ perfusate. The release of s-PECAM was paralleled by a significant reduction of PECAM-1 (CD31) expression in the lungs at the end of 6h of EVLP (**Figure 9C**). All these changes were abrogated in the EVLP_HS_ group (**Figures 9A–C**). Moreover, EVLP_37°C_ lungs released the adhesion molecules sE-selectin and s-ICAM-1 in the perfusate in significantly greater amounts than the EVLP_HS_ lungs (**Figures 9D–E**).

## 4 Discussion

Lung I/R injury following lung transplantation may precipitate primary graft dysfunction, with significant short- and long-term consequences (23). A hallmark of I/R injury is endothelial damage and dysfunction, resulting in the loss of endothelial barrier function, high permeability edema, vasoactive imbalance, procoagulant activity and the recruitment and activation of inflammatory leukocytes (4–6). The search for novel strategies to protect the endothelium appears therefore crucial to improve LTx outcomes (4). In this respect, the development of EVLP has represented a major breakthrough, by permitting the evaluation of endothelial dysfunction prior to LTx (24, 25), as well as for potential therapeutic interventions (16). In a recent study, we provided evidence that the transient application of a mild heat stress during a relatively short EVLP protocol (3h) reduced innate immune signaling, oxidative stress and epithelial damage in rat lung grafts damaged by warm ischemia (15). Our present results significantly extend these observations, by showing that therapeutic heat application provides significant protection against the endothelial damage and dysfunction developing over an extended period (6h) of EVLP.

Transient (30 min) heating at 41.5° after 1h EVLP promoted the expression of two key inducible heat shock proteins, namely HSP70 and HSP27, starting about 2h after heat exposure and maintained until the end of the 6h reperfusion (**Figure 2**). By contrast, heat stress did not enhance the expression of constitutive Hsc70, but prevented its downregulation, as noted in non-heated lungs after 6h. It is noteworthy that the activity of both HSP70 and Hsc70 is regulated by ATP binding and hydrolysis (26). As we did not measure lung tissue ATP, we cannot determine whether possible changes in ATP content during EVLP might have influenced their activities. Previous investigations revealed that both HSP70 and HSP27 could reduce endothelial damage and permeability in non-pulmonary vessels by maintaining the integrity of cell junction proteins (27) and by preventing actin polymerization (28). Here, we found that heat-treated lungs displayed a marked reduction of lung edema after 6h reperfusion, an effect translating into preserved oxygenation capacity and static lung compliance (**Figure 3**). Since the formation of lung edema during reperfusion primarily reflects the increase of endothelial permeability (29, 30), this observation points to significant protection of the endothelial barrier function by heat therapy, an assertion further supported by the decrease in perivascular edema at histological evaluation (**Figure 4**). In addition, we may also consider the role of reduced capillary hydrostatic forces (31), as suggested by the attenuated increase of PA pressure during reperfusion of heat-treated lungs. It is here worth to mention that, owing to the constant flow conditions in our model, the increase of PA pressure during EVLP was entirely related to an increase in PVR, the latter being significantly blunted by heat therapy (**Figure 5**). It has been shown that an increase in PVR directly correlates with endothelial dysfunction during experimental LTx (32), and that the degree of PVR increase during EVLP is associated with the severity of PGD after LTx (33, 34). Collectively, these arguments support that heat therapy significantly attenuated endothelial dysfunction and the increased endothelial permeability developing over the 6h of EVLP.

The endothelial barrier function depends on the integrity of inter-endothelial cell connections, comprising adherens (primarily VE-cadherin) and tight (primarily occludins and claudins) junction proteins, connected to the actin cytoskeleton through interactions with catenins, zonula occludens proteins and cingulin (22). Opening of these intercellular junctions precipitates high permeability edema, as occurs during inflammation or ischemia-reperfusion. Factors increasing endothelial permeability, such as inflammatory mediators, VEGF and thrombin, trigger signaling cascades leading to the remodeling of endothelial junctions and the inhibition of VE-cadherin adhesive function, largely related to phosphorylation of VE-cadherin at Tyr^685^ by Src protein tyrosine kinase (22). Src itself is activated through Tyr^419^ phosphorylation, whereas phosphorylation at Tyr^529^ (Tyr^530^ in humans) leads to decreased activity (35). Our findings indicate that such signaling events were, indeed, triggered after 6h EVLP, as indicated by intense Src Tyr^419^ phosphorylation, concomitant to Tyr^529^ dephosphorylation, associated with the phosphorylation of VE-cadherin at Tyr^685^ (**Figure 6**). Most importantly, these events were abrogated when lungs were subjected to transient heating, confirming the significant potential of this intervention to prevent the deterioration of endothelial barrier function during prolonged reperfusion.

A possible mechanism underlying these observations may rely in a reduction of oxidant-dependent Src kinase activation (36), in view of the protective actions of heat stress against oxidant-dependent processes (15, 37, 38). Notably, Src is very sensitive to activation by peroxinitrite, a potent oxidant species formed from nitric oxide and superoxide (39), which has been associated with the development of endothelial dysfunction in various conditions, including I/R injury (39). We evaluated endothelial peroxynitrite formation in lung sections after 6h EVLP, using immunofluorescent detection of 3-nitrotyrosine, a stable footprint of peroxinitrite, and of the endothelial marker CD31 (**Figure 7**). In the EVLP_37°C_ group, the endothelial lining of alveolar vessels was thickened and intensely positive for 3-NT, contrasting with an attenuated 3-NT staining in the thinner alveolar vessels of heat-treated lungs. While these findings indicate that heat stress reduced vascular peroxinitrite generation, the latter was, nevertheless, not abrogated. Thus, additional mechanisms must be considered to explain the profound reduction of Src kinase activation and VE-cadherin phosphorylation following heat application.

One such mechanism could be the direct inhibition of Src activity by HSP70, as reported in cancer cells (40). Furthermore, the inflammatory cytokines TNFα and IL-1β are potent initiators of increased endothelial permeability (22), an effect mediated, in part, by the activation of Src-family protein kinases (41–43). Copious amounts of both TNFα and IL-1β were released in the perfusate of EVLP_37°C_ lungs over the 6h of observation (**Figure 8**), which is consistent with data reported by us (17, 20) and other investigators (44, 45). The release of cytokines was largely reduced by heat stress, confirming our recent studies showing that heat therapy lessened innate immune activation during EVLP and LTx through the inhibition of NF-κB and the inflammasome (15). Taken together, these results suggest that heat stress decreased Src kinase activation leading to VE-cadherin phosphorylation through at least two distinct mechanisms, namely the reduction of endothelial peroxinitrite formation, and the blunted expression of inflammatory cytokines.

We then assessed the influence of heat stress on the release of the endothelial biomarkers PECAM-1, vWF, E-selectin and ICAM-1 during EVLP. PECAM-1 (CD31), is a transmembrane protein critical for the maintenance of the normal endothelial barrier, detectable in plasma as a soluble truncated protein (sPECAM) upon cleavage from damaged endothelial cells (46). VWF, constitutively expressed and stored in endothelial cells, plays important roles in endothelial adhesion and hemostasis, and is released extracellularly under conditions of endothelial damage and activation (47). E-selectin and ICAM-1 are inducible adhesion molecules expressed by the endothelium upon activation by inflammatory cytokines (48). We found that sPECAM increased substantially in the perfusate of non-heated lungs, an effect associated with the downregulation of lung PECAM-1 expression, implying that severe endothelial cell injury resulted in the shedding of PECAM-1 into the extracellular milieu. Similarly, the perfusate levels of vWF, E-selectin and ICAM-1 steadily increased over time, further confirming significant endothelial damage and activation during reperfusion. At variance with these findings, heat-treated lungs released considerably less vWF and sPECAM, while maintaining tissue PECAM-1 expression, indicating better preservation of their endothelial integrity. Furthermore, the accumulation of E-selectin and ICAM-1 in the perfusate was also significantly attenuated by heat-stress, albeit in a lesser proportion than sPECAM and vWF, which could reflect a greater functional endothelium in heat-treated lungs, as suggested by their greater PECAM-1 expression.

Our study has several limitations. The first one is that we did not evaluate endothelial function after transplantation. This was related to the experimental design, which aimed to address the effects of heat stress during an extended period of EVLP. Indeed, the progressive amplification of endothelial damage during prolonged reperfusion may limit the duration during which lung grafts can be maintained in an EVLP system. Our work was therefore designed to determine if therapeutic heat stress allowed to safely extend the duration of EVLP. In addition, due to the severe edema noted after 6h in lungs from the EVLP_37°C_ group, we could not envision their transplantation into recipient animals. A second limitation is that we performed several measurements in whole lung tissue extracts but not directly in endothelial cells. Future studies will therefore be needed using endothelial cells isolated from the lungs at different stages of EVLP, to address their specific response to heat stress in terms of heat shock protein expression and modulation of various molecular pathways. Also, physiological studies in isolated pulmonary vessels will permit to address the influence of heat stress on vascular reactivity and notably on endothelial-dependent vasodilation. Notwithstanding this limitation, our findings that heat stress reduced the phosphorylation of VE-cadherin, a junction protein exclusively expressed by endothelial cells, and prevented the downregulation of the endothelial antigen CD31 (PECAM-1), point to the direct influence of heat stress on endothelial cells. Finally, we acknowledge that the number of observations was relatively small, with inherent limitations to the statistical analyses.

In conclusion, we demonstrate that a transient heat stress, applied on rat lungs damaged by warm ischemia, protects from endothelial damage and dysfunction during prolonged reperfusion. This conclusion is supported by the significant attenuation of lung edema and physiological deterioration, the suppression of Src kinase activation and VE-cadherin phosphorylation, the reduction of endothelial peroxinitrite generation, the prevention of endothelial PECAM-1 shedding and release of von Willebrand factor, as well as by the downregulated expression of E-selectin and ICAM-1. Collectively, these findings support the concept that ex-vivo heat stress may represent a promising strategy to protect the endothelium from the damaging effect of reperfusion, thereby allowing to safely extend the duration of EVLP.

## Data availability statement

The original contributions presented in the study are included in the article/**Supplementary Material**. Further inquiries can be directed to the corresponding author.

## Ethics statement

The animal study was approved by Direction générale de l’agriculture, de la viticulture et des affaires vétérinaires (DGAV) de l’Etat de Vaud. The study was conducted in accordance with the local legislation and institutional requirements.

## Author contributions

RP: Conceptualization, Data curation, Formal Analysis, Investigation, Writing – original draft, Writing – review & editing. AD: Conceptualization, Data curation, Formal Analysis, Investigation, Writing – original draft, Writing – review & editing. MA: Data curation, Formal Analysis, Investigation, Writing – review & editing. JL: Data curation, Formal Analysis, Investigation, Writing – review & editing. HR-C: Data curation, Formal Analysis, Investigation, Writing – review & editing. LL: Conceptualization, Data curation, Formal Analysis, Funding acquisition, Project administration, Writing – original draft, Writing – review & editing. TK: Conceptualization, Data curation, Formal Analysis, Funding acquisition, Project administration, Writing – original draft, Writing – review & editing.
