# Supplementary material for: Transient heat stress protects from severe endothelial damage and dysfunction during prolonged experimental ex-vivo lung perfusion
Source: Front Immunol. 2024 May 14;15:1390026. doi: 10.3389/fimmu.2024.1390026 (PMC11130382; doi:10.3389/fimmu.2024.1390026)
Supplement: Supplementary file 1 [file DataSheet_1.pdf]

Supplementary Table S1. List of antibodies and ELISAs used in the study

**Antibodies (western blots)**	**References**	**Supplier**
Phospho-Tyr^{419} SRC	# 44-660G	Invitrogen
Phospho-Tyr^{259} SRC	# 44-662G	Invitrogen
SRC	# AHO1152	Invitrogen
Beta-actin	# MA5-15739	Invitrogen
Phospho-Tyr^{685}VE-cadherin	# PA5-143661	Invitrogen
VE-cadherin	# 36-1900	Invitrogen
Mouse-HRP (secondary)	# 115-035-174	Jackson ImmunoResearch
Rabbit-HRP (secondary)	# 211-032-171	Jackson ImmunoResearch
**Antibodies (immunofluorescence)**		
3-nitrotyrosine	A21285	Invitrogen
CD31 (PECAM-1)	AF3628	R&D Systems
α-goat Alexa 555 (secondary)	A21482	Invitrogen
α-rabbit Alexa 647 (secondary)	A31573	Invitrogen
**ELISAs**		
Rat HSP70	ADI-EKS-700B	Enzo Life Sciences
Rat HSPB1/HSP27	LS-F4101	LifeSpan BioSciences
Rat HSC70	LS-F34032	LifeSpan BioSciences
Rat von Willebrand Factor	LS-F21631	LifeSpan BioSciences
Rat IL-1 beta/IL-1F2 DuoSet	DY501	R&D Systems
Rat TNF-alpha DuoSet	DY510	R&D Systems
Rat sICAM-1 Quantikine®	RIC 100	R&D Systems
Rat sPECAM-1	CSB-E08134r	Cusabio Technology LLC
Rat PECAM-1	RTFI00178	Assay Genie
Rat sE-Selectin	CSB-E07996r	Cusabio Technology LLC
