# Supplementary material for: Transient heat stress protects from severe endothelial damage and dysfunction during prolonged experimental ex-vivo lung perfusion
Source: Front Immunol. 2024 May 14;15:1390026. doi: 10.3389/fimmu.2024.1390026 (PMC11130382; doi:10.3389/fimmu.2024.1390026)
Supplement: Supplementary file 2 [file DataSheet_2.pdf]

Supplementary File S2. Fiji script for the analysis of immunofluorescence images.

```
idOrig = getImageID();

roiManager("reset");
run("Set Measurements...", "area area_fraction display redirect=None decimal=3");

// Set working images
selectImage(idOrig);
run("Duplicate...", "duplicate");
idROIOrig = getImageID();

selectImage(idROIOrig);
run("Duplicate...", "duplicate channels=1");
idECMask = getImageID();
run("Duplicate...", "duplicate");
idECShow = getImageID();

selectImage(idROIOrig);
run("Duplicate...", "duplicate channels=2");
idNitroMask = getImageID();

// Set EC region // Values threshold must be adapted with C-
selectImage(idECMask);

run("Gaussian Blur...", "sigma=4");
setAutoThreshold("Default dark");
run("Threshold...");
setThreshold(35,255)
```

```
setOption("BlackBackground")
run("Convert to Mask");
run("Despeckle");
run("Dilate");
run("ROI Manager...");
selectImage(idECShow);
setBatchMode("show");
selectImage(idECMask);
setBatchMode("show");
// Program stopped to check Mask and Save if wanted
waitForUser;
// clic OK when done;
run("Create Selection");
roiManager("Add");
roiManager("Select", 0);
roiManager("Rename", "CD31 Area");


// Set Nitro Area
selectImage(idNitroMask);
run("Gaussian Blur...", "sigma=4");
setAutoThreshold("Default dark");
run("Threshold...");

// Measurement
selectImage(idNitroMask);
roiManager("Select", 0);
roiManager("Measure");
close("*");
```
